# Research on Internal Damage Identification of Wire Rope Based on Improved VGG Network

**DOI:** 10.3390/e26070531

**Published:** 2024-06-21

**Authors:** Pengbo Li, Jie Tian

**Affiliations:** 1 School of Mechanical, Electronic and Information Engineering, China University of Mining and Technology (Beijing), Beijing 100083, China; 2 Key Laboratory of Intelligent Mining and Robotics, Ministry of Emergency Management, Beijing 100083, China

**Keywords:** improved VGG model, the joint samples uniformly distributed cross-entropy, STFT method, quantitative identification, internal damage of wire rope

## Abstract

In order to solve the problem of great difficulty in detecting the internal damage of wire rope, this paper proposes a method to improve the VGG model to identify the internal damage of wire rope. The short-time Fourier transform method is used to transform the wire rope damage signal into a time-frequency spectrogram as the model input, and then the traditional VGG model is improved from three aspects: firstly, the attention mechanism module is introduced to increase the effective feature weights, which effectively improves the recognition accuracy; and then, the batch normalization layer is added to carry out a uniform normalization of the data, so as to make the model easier to converge. At the same time, the pooling layer and the fully connected layer are improved to solve the redundancy problem of the traditional VGG network model, which makes the model structure more lightweight, greatly saves the computational cost, shortens the training time, and finally adopts the joint-sample uniformly distributed cross-entropy as the loss function to solve the overfitting problem and further improve the recognition rate. The experimental results show that the improved VGG model has an identification accuracy of up to 98.84% for the internal damage spectrogram of the wire rope, which shows a good identification ability. Not only that, but the model is also superior, with less time-consuming training and stronger generalization ability.

## 1. Introduction

Wire rope is a kind of rope twisted by multiple strands of steel wire with high strength, wear resistance, and corrosion resistance. It is usually used in the fields of mine hoisting, haulage, ropeways, ship towing, and other fields and is a common engineering material [1,2,3]. Due to the harsh working environment of the mine, in the process of long-term use, it is inevitable that different damages will occur under the long-term influence of tensile bending, alternating loads, environmental corrosion, and other factors, resulting in safety accidents. When using steel wire rope, attention needs to be paid to its regular damage detection to ensure that it will not be broken or damaged in the process of use and to protect the safety and service life of steel wire rope [4,5,6,7]. Therefore, how to detect wire rope damage accurately and efficiently is the focus of the current research field on wire rope.

In recent years, many scholars have conducted a lot of research on wire rope damage recognition using artificial intelligence methods [8,9,10]. Shiliang et al. [11] used the nearest neighbor (NN) algorithm to quantitatively identify the wire rope damage and verified it by comparing it with the backpropagation (BP) neural network. Xi, L. et al. [12] proposed a wire rope broken wire recognition method based on the fusion of magnetic infrared information. Juwei, Z et al. [13] built a magnetic memory detection platform for wire rope under weak magnetic field excitation, obtained the fused magnetic memory signals, and at the same time, the defective image was subjected to feature extraction, and the wire rope damage was identified by the GWO-SVM algorithm. Not only that, Juwei, Z. et al. [14] used principal component analysis to reduce the dimensionality of the magnetic leakage features of the wire rope and input the extracted features into a back propagation network for quantitative identification. Qiang, Y. et al. [15] used the HOG algorithm to extract the wire rope damage features and used a combination of BP neural networks and support vector machines for quantitative identification of wire rope wire-break damage.

However, at present, most scholars mainly focus on external damage identification research; wire rope internal damage research is less, but wire rope internal damage, due to its hidden nature, is often one of the most important causes of safety accidents; therefore, more cannot be ignored. At the same time, most of the current wire rope identification methods use artificial extraction of features for damage identification; their cost is high, and a certain amount of a priori knowledge of the intervention is prone to misjudgment, so there is an urgent need for an unsupervised adaptive identification of the wire rope damage. At this time, deep learning theory entered the sights of many scholars.

Deep learning methods have been developing rapidly in recent years and have received extensive attention in many fields due to their good generalization ability and self-adaptability [16,17,18,19,20]. Among them, Yu, H. et al. [21] proposed a novel deep learning-based defect detection method for steel plates that used convolutional neural networks, multi-feature fusion networks (MFN) to extract the steel plate damage features, and area suggestion networks (MFN) combined with classifiers to detect the damage. Weigang, W. et al. [22] proposed a deep convolutional neural network-based fault diagnosis method for rotating machines. Combining the sensory field of DCNN with the vibration signals of the rotating machine, the fault diagnosis of the rotating machine was achieved. Zan, W. et al. [23] proposed an AUTO-CNN network for bearing fault diagnosis, which used hierarchical coding strategy and spatial exploration strategy to optimize the structure of CNN, and the fault diagnosis of bearings was achieved. Yunlong, W. et al. [24] proposed, based on the electromechanical actuator, a deep learning adaptive identification method that introduces the joint maximum mean difference into the convolutional neural network to achieve the diagnosis of electromechanical actuator faults. Xu, L. et al. [25] proposed a long-short memory neural network to predict the indentation of metal coating materials, which is conducive to the interpretation of the elasticity and plasticity behaviors of the coating materials from the perspective of indentation measurement.

Although deep learning in several fields has achieved certain results with good generalization ability and is adaptive, in the case of wire rope damage recognition, there are still two major problems. Firstly, the internal damage of the wire rope is usually small, and the damage samples are weak, which brings certain difficulties to the deep model recognition, and secondly, due to the special working environment of mining wire rope, in order to protect its safety, the detection model needs to be accurate and efficient, while the traditional deep model is complex with more free parameters, which consumes a lot of time and is less efficient when carrying out wire rope damage recognition.

Therefore, in order to solve the problem of the high difficulty and low recognition efficiency of internal damage recognition of wire rope, this paper proposes an improved VGG model based on attention mechanisms for internal damage recognition of wire rope. Firstly, the STFT method is used to transform the wire rope damage signal into a time-frequency spectrogram as the model input, and then the attention mechanism is introduced to increase the weight of the effective features to improve the recognition rate. At the same time, the overall structure of the VGG model is improved to reduce the training parameters and training time. Finally, the joint sample uniformly distributed cross-entropy is used as the loss function, which solves the overfitting problem and further improves the recognition rate, and the effectiveness of this paper’s method is verified by wire rope damage experiments.

The chapters of this paper are organized as follows: Section 2 describes the process of spectrogram acquisition during internal damage to wire rope. Section 3 introduces the improvement method of the VGG model. Section 4 described the SE-WRVGG model experimental validation and result analysis. Section 5 concludes the article.

## 2. Spectrogram Acquisition during Internal Damage of Wire Rope

The internal damage signal of the wire rope is collected by the leakage magnetic detection method, and the time-frequency map of the internal damage of the wire rope is generated by the STFT [26] method, which is a technique commonly used for the spectral analysis of time-varying signals with excellent time-frequency resolution and can more accurately capture the transient characteristics of the signal. In addition, STFT can process signals in real time, which is suitable for real-time monitoring. More importantly, the STFT method can adjust the time-frequency resolution according to the need to adapt to the analysis of different signal characteristics, so it is considered a flexible and efficient time-frequency analysis method.

In this study, the sampling frequency of the wire rope damage signal is 2000 Hz, and its sampling process needs to be collected online in real time. Due to the difference in damage frequency between different damages, the STFT method can match the characteristics of the internal damage signal of the wire rope. After the STFT method, the generated internal damage image of the wire rope has time-frequency domain double features, which can comprehensively show the internal damage of the wire rope to determine the degree of damage more intuitively within the wire rope.

In summary, the acquisition of wire rope damage signals by the leakage magnetic detection method and the time-frequency analysis combined with the STFT method can provide a more accurate understanding of the damage inside the wire rope. The method not only provides the ability to analyze with high time-frequency resolution but can also monitor the signal in real time and flexibly adjust the resolution.

### 2.1. Wire Rope Internal Damage Magnetic Flux Leakage Detection Principle

**Figure 1:**
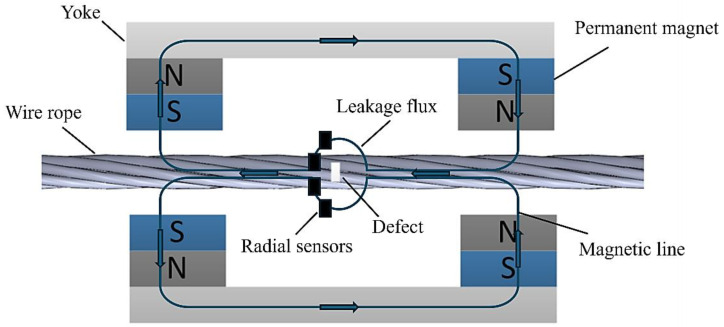
Magnetic leakage detection of internal damage to the wire rope.

Wire rope internal damage detection is the use of magnets on the wire rope to fully magnetize it through the detection of changes in the magnetic induction strength of the wire rope to determine whether there is damage within the wire rope. Figure 1 is the detection principal diagram of the internal damage to the wire rope.

If there is no defect inside the wire rope, the magnetic induction line is parallel to the wire rope. If there is a defect in the wire rope, the magnetic induction will be deflected to leak out of the surface of the wire rope, forming a magnetic flux leakage. At this time, through the detection of the radial sensors to collect the damage signal, we can determine the type and location of the defect.

### 2.2. Wire Rope Damage Signal Acquisition and Time-Frequency Spectrogram Imaging

**Figure 2:**
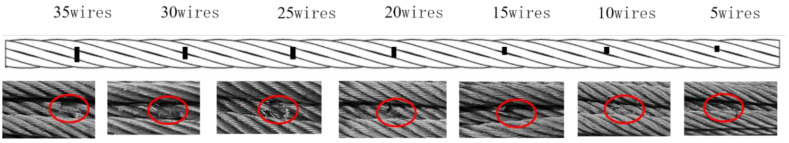
Specific location and actual damage on the wire rope.

In this paper, an experimental study was carried out using a 6 × 19 wire rope with a diameter of 30 mm. Seven broken wire damages were produced on the wire rope, and the numbers of broken wires were 5, 10, 15, 20, 25, 30, and 35 wires, respectively. The specific location of the damage on the wire rope and the actual damage are shown in Figure 2. The picture of the internal damage of the wire rope in Figure 2 is obtained by photographing the wire rope after loosening the strands and after the internal damage is revealed.

**Figure 3:**
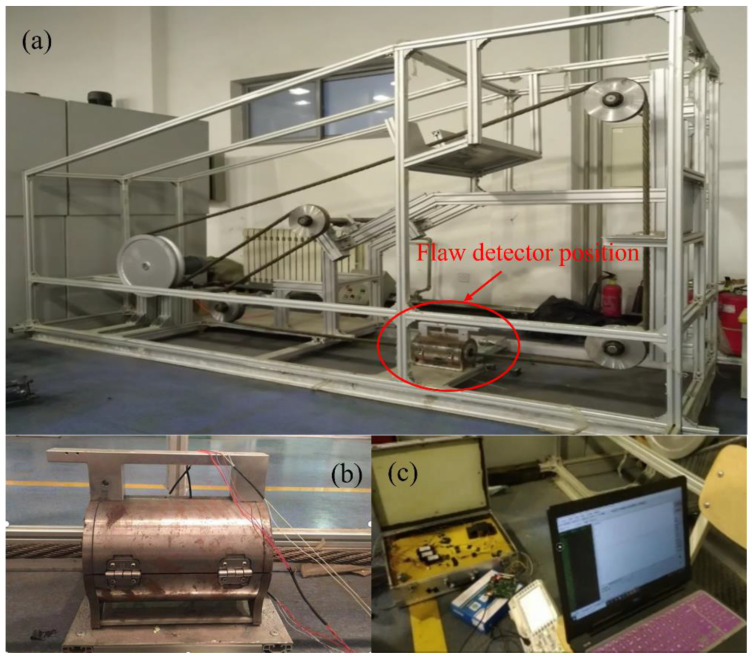
Diagram of the experimental setup (**a**) Experimental platform; (**b**) Flaw detector; and (**c**) Acquisition system device.

The wire rope testing experimental bench is shown in Figure 3a. With internal damage to the wire rope through the experimental overall bracket installed on the experimental bench and through the motor to control the running speed of the wire rope, the flaw detector is installed in the designated location of the wire rope internal damage detection, and the flaw detector output signal through the signal acquisition device pre-processing is transmitted to the signal acquisition system in the computer testing platform for display and signal preservation. Flaw detector and acquisition systems are shown in Figure 3b,c.

After the signal acquisition is completed, the data is converted into time-frequency spectral images. The STFT method is commonly used in the time-varying signal spectrum analysis, and the characteristics of the wire rope damage signal match the time-frequency spectral image of different damage signals. There are obvious differences. At the same time, after the method of processing the image of the internal damage to the wire rope has time-frequency domain double features, the existence of the damage inside the wire rope presents a complete image display. At the same time, the image of the internal damage to the wire rope, processed by this method, has double features in the time and frequency domain, which can present the complete image display of the damage existing inside the wire rope and the internal damage of the wire rope can be judged visually.

Its working principle is that, before applying the Fourier transform, the signal is multiplied by a very short time window function. This window function intercepts the signal, allowing the original non-smooth signal to be analyzed as a smoothed signal. Finally, the time window is slid, and the spectral combination of each window function signal is the time-frequency spectrogram of the whole wire rope damage signal. The expression is as follows:

(1) $$STFT(t,f)=\sum_{u=0}^{L-1}s(u)g(u-t)e^{-j2\pi fu}$$

where STFT(t,f) is the time-frequency spectrum of signal s(u), t denotes time, f is frequency, and s(u) denotes the original signal at the moment of u. The sliding window function of the original signal at the moment u is g(u−t), and L is the length of the window function.

**Figure 4:**
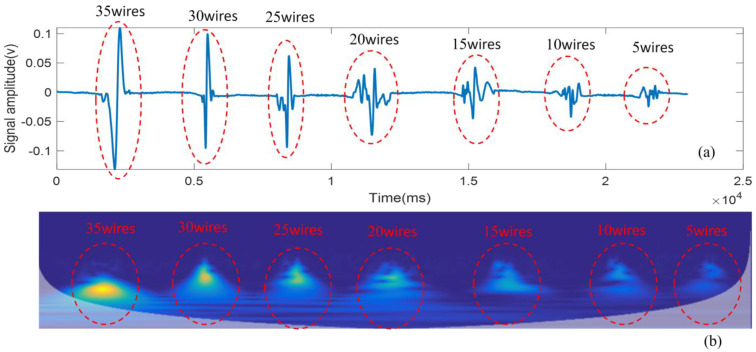
Converted time-frequency spectra of the damage signals inside the wires rope (**a**) for the original signals (**b**) converted time-frequency spectra.

The time-frequency spectrogram of the transformed internal damage signal of the wire rope using the STFT method is shown in Figure 4.

As can be seen from Figure 4, the internal damage depth of the wire rope changes, resulting in damage to the magnetic signal that also changes accordingly. When the internal damage depth increases, the amplitude of the damaged magnetic signal also increases. Using the STFT method of wire rope internal damage signal after the transformation of the time-frequency spectrogram, based on the energy (the middle of the yellow part), different internal damage of the wire rope is characterized; at the same time, the internal damage energy of the 35 wire is the largest, and the internal damage energy of the 5 wire is the smallest. According to the characteristics of this feature, it can be used as a basis for the identification of internal damage to the wire rope.

## 3. SE-WRVGG Model of Wire Rope Internal Damage Detection Improvement Method

### VGG Model

The VGG [27] network is a classical deep convolutional neural network architecture proposed by a team of researchers at the University of Oxford. There are several important reasons for using VGG networks:

Deep network structure: The VGG architecture is known for its depth, using smaller 3 × 3 convolutional kernels and a deeper network structure that helps to learn more complex and abstract features. By stacking multiple convolutional and pooling layers, the VGG network can better capture features in the data.

Good performance: The VGG architecture performs well on computer vision tasks such as image classification, achieving excellent performance on multiple datasets. Its simple yet effective design makes VGG networks easy to train and tune for a variety of recognition tasks.

Easy to understand and implement: VGG networks have a clear and simple structure, using only basic convolution and pooling operations, with no complex structures or modules. This simplicity makes VGG networks easy to understand and implement, as well as easy to modify and extend.

Small sample learning: by fine-tuning the pre-trained VGG model, good results can be achieved on small datasets, saving training time and resources.

**Figure 5:**
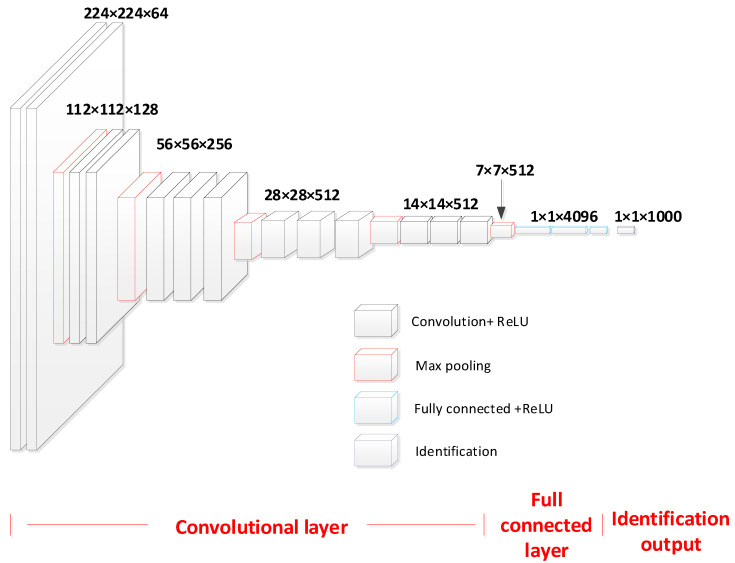
Structure of traditional VGG network.

Overall, due to the advantages of depth, performance, and simplicity of the VGG network, its overall structure is very suitable for the direction of damage recognition, and the network structure is shown in Figure 5. Therefore, this paper adopts the VGG network as the main architecture and improves it.

**Figure 6:**
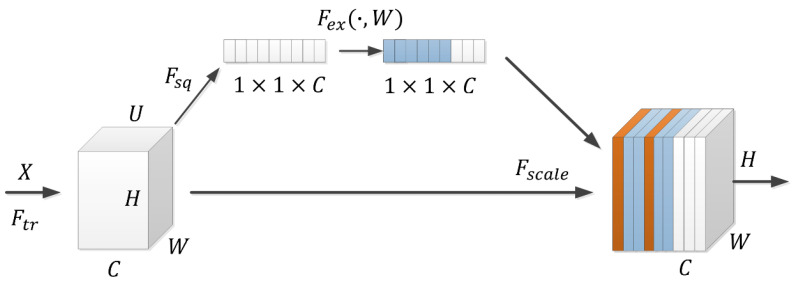
SE module schematic diagram.

In this paper, improvements are made to the VGG model to make it the SE-WRVGG model for internal damage identification of wire ropes, as follows:

(1) Attention mechanisms:

Firstly, the SE network is embedded in the new model, which solves the dependency problem between channels by learning the global information, enhances the valuable feature information by autonomous selection, reduces the interference of useless information, and re-adjusts the occupancy ratio of the features to enhance the quality of the training features. It solves the problem that VGG16 only deals with picture information from the plane dimension and ignores the importance of information processing in the channel dimension. The SE network module is mainly composed of four parts: transformation operation ($F_{tr}$), squeezing operation ($F_{sq}$), excitation operation ($F_{ex}$), and proportion operation ($F_{\mathrm{scale}}$). The schematic diagram of the SE module based on the attention mechanism is shown in Figure 6.

Conversion operation: The transformation operation is a standard convolution operation that converts the input to a three-dimensional matrix, i.e., C feature maps of size H×W, as shown in $F_{tr}$ in Figure 5. Its transformation expression is shown below:

(2) $$F_{tr}:X\to U,U\in R^{W\times H\times C}$$

where X is the input, U is the transformed 3D matrix, and *W*, *H*, and *C* are the 3D vectors of the matrix, respectively.

Squeezing operation: After the data conversion is completed, the feature map is compressed so that the C feature maps become a 1×1×C array of real numbers, i.e., the squeezing operation. Corresponding to the $F_{sq}$ operation in Figure 5, it is expressed as follows:

(3) $$Z_{c}=F_{sq}(u_{c})=\frac{1}{W\times H}\sum_{i=1}^{W}\times \sum_{j=1}^{H}u_{c}(i,j)$$

where $F_{sq}$ is the squeezing operation, $u_{c}$ is the channel feature of the feature map u, i,j acts as a counting function, and $Z_{c}$ is the result after feature compression.

Incentive operation: The SE module captures the channel dependency effect through the excitation operation; that is, after the pooling layer is compressed, the SE module is used to assign the feature weights, and the features with larger weights can be mapped efficiently when entering, e.g., the fully connected layer, which is expressed by the following formula:

(4) $$s=F_{ex}\left(z,W\right)=X_{n\times i}W_{j\times o}+b$$

where s is the weight of the feature map; $F_{ex}$ is the excitation operation; z is the result after feature compression; X is the samples to be learned by the model; $X_{i}$ would denote the ith sample to be learned by the model; W is the parameter to be learned by the model; $W_{j}$ denotes the weights of all the input neurons up to the jth output neuron; b is the o-dimensional vector bias; and n is the number of rows in the input vector.

Proportional operation: The scale operation corresponds to $F_{\mathrm{scale}}$ in Figure 5, i.e., the operation is performed on the original U after obtaining s, which is calculated as follows:

(5) $${\overset{\sim }{X}}_{c}=F_{\mathrm{scale}}(u_{c},S_{c})=S_{c}\cdot u_{c}$$

where $F_{\mathrm{scale}}(u_{c},S_{c})$ is the corresponding channel product between $u_{c}$ and $S_{c}$; $u_{c}$ denotes the two-dimensional matrix of the cth channel in U, and $S_{c}$ is a value in the output s from the previous step.

(2) Improved network structure:

In this paper, the BN batch normalization layer is added to the feature layer, which can process the data uniformly, make the data more regular, solve the problem of internal covariate offset, and facilitate model operation and convergence. The BN layer is normalized by calculating the mean and variance of all the pixel points in the feature map of a single channel to obtain the determined value that can represent a single channel, and the formula is as follows:

(6) $$\mu =\frac{1}{m}\sum_{i=1}^{m}x_{i}$$

(7) $$\sigma^{2}=\frac{1}{m}\sum_{i=1}^{m}{\left(x_{i}-\mu \right)}^{2}$$

(8) $$X_{i}=\frac{x_{i}-\mu }{\sqrt{\sigma^{2}+\epsilon }}$$

(9) $$y_{i}=\gamma \times X_{i}+\beta$$

where m is a randomly selected small batch of data, $x_{i}$ is a feature of the input, μ is the mean, σ is the variance, γ and β are learning parameters, and ϵ is a regular parameter.

In order to solve the problem of too many parameters and the complexity of computation in the traditional VGG network, in this paper, the first fully connected layer and the second fully connected layer of the model are removed, and the maximum pooling layer is replaced by the global average pooling layer. The overall network structure of the convolutional layer, the global average pooling layer, and one fully connected layer are finally obtained, which solves the problem of too many parameters in the original network to a certain extent and reduces the training time and computation amount. Among them, the global average pooling layer is used to replace the maximum pooling layer because the global average pooling layer corresponds to each channel of the feature map to a point in the global average pooling layer, thus giving meaning to the channel. At the same time, the structure requires negligible computation and does not need any hyperparameters to be adjusted, which can alleviate the redundancy problem arising from the use of the maximal pooling layer to a certain extent and greatly increase the anti-overfitting effect. At the same time, because the global pooling layer can play the role of the fully connected layer in the network, i.e., the input multi-dimensional features into a unit, this paper removes the first fully connected layer and the second fully connected layer to lighten the network structure, but the addition of the global average pooling layer is not a complete replacement for the fully connected layer, there is no learning parameter in the global average pooling layer, but is combined with the convolutional layer to complete the extraction of the features, transformation, and dimensionality reduction. Therefore, the retention of a maximum pooling layer cannot completely replace the fully connected layer and reduce dimensionality. Therefore, retaining a fully connected layer, combined with the actual problem, can further improve the recognition accuracy. Its functional expression is

(10) $$y^{c}=\frac{1}{n}\sum_{i=1}^{s}\;x_{i}^{c}$$

where $y^{c}$ is the global average of the cth channel of the input feature map; n is the number of features per channel; and $x_{i}^{c}$ is the ith feature value of the cth channel.

(3) The joint samples uniformly distributed cross-entropy:

In this paper, cross-entropy is used as the loss function. As the result of its classification calculation is non-zero or one, it will inevitably cause overfitting, and the threshold cannot be set in practice. In order to prevent overfitting, this paper proposes a cross-entropy loss method with a uniform distribution of joint samples. The cross-entropy loss $L_{mean}$ for a uniform distribution of samples is

(11) $$L_{mean}=-\frac{1}{m}\sum_{i=1}^{m}\;log\frac{e^{X^{T}W_{j}}}{\sum_{j=1}^{k}\;e^{X^{T}W_{j}}}$$

where m denotes the number of training samples; $W_{j}$ is the jth parameter of the network model; and k is the number of identified classifications.

The sample cross-entropy loss is united with the uniformly distributed cross-entropy loss thus as the total loss, which is calculated as follows:

(12) $$L=(1-\lambda {)}_{S}+\lambda_{mean}$$

where $(1-\lambda {)}_{S}$ denotes the sample cross-entropy loss; $\lambda_{mean}$ denotes the uniformly distributed cross-entropy loss; and λ denotes the coefficient used to balance the two loss functions.

**Figure 7:**
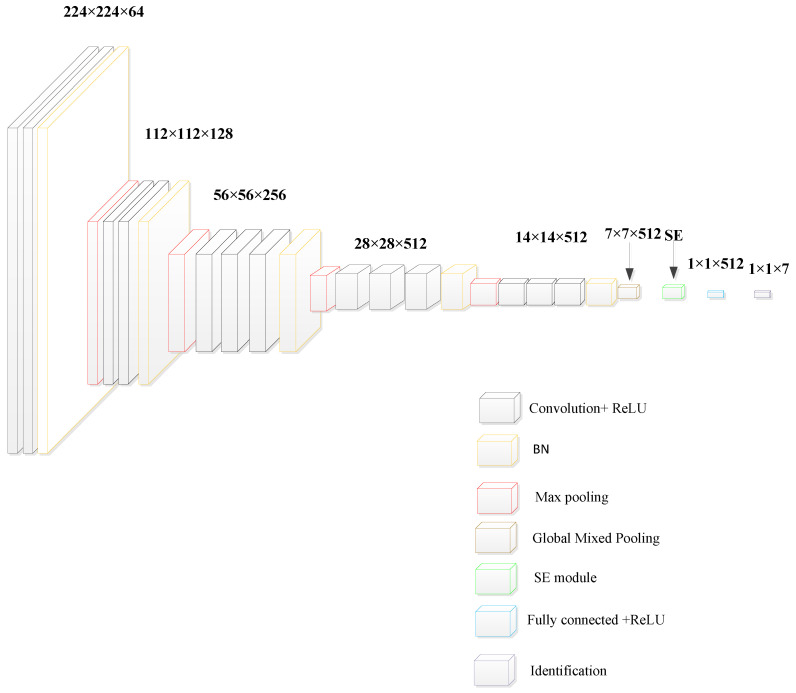
Overall structure of SE-WRVGG network.

In summary, the overall structure of the SE-WRVGG network is shown in Figure 7.

## 4. SE-WRVGG16 Model Experiments and Analysis of Results

**Figure 8:**
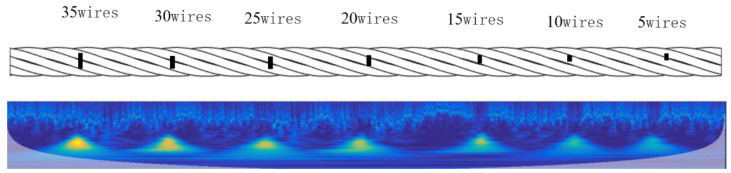
Images of some of the experimental datasets.

(1) Experimental data: The experimental data comes from the experimental wire rope damage detection data. The dataset includes seven kinds of internal damage data of the wire rope, each kind of damage dataset 500, a total of 3500 time-frequency map samples for experimental testing. At the same time, in the process of training, we follow the ratio of the training set and the test set of 8:2 for training. Figure 8 shows some of the experimental dataset images.

(2) Experiments and parameter settings: The experiments are trained using the Keras framework. The training model is based on the SE-WRVVG algorithm model, and the small batch stochastic gradient descent with momentum factor is used to train the network. The initial learning rate is 0.001, the number of iterations is 20,000, and the activation function is Relu. The graphics card is MSI 4060, and according to the performance of the graphics card and the characteristics of the small batch, the momentum factor is 0.9, the weight decay is 0.005, and the regularization coefficient is 0.0005.

(3) Evaluation index: In this paper, the model is evaluated using accuracy, precision, recall, and the F1 value. Accuracy (Acc) denotes the proportion of correctly predicted samples to the total samples; precision (Pre) denotes the proportion of samples in which both the actual category and the predicted category are positive to the proportion of all samples in which the predicted category is positive; recall (Rec) denotes the proportion of samples in which both the actual category and the predicted category are positive to the proportion of all samples in which the actual category is positive; and the F1 value is the weighted summed average of the accuracy and the recall. The metrics are calculated as shown below:

(13) $$Acc=\frac{TP+FN}{TP+TN+FP+FN}\times 100\%$$

(14) $$\mathrm{Pre}=\frac{TP}{TP+FP}\times 100\%$$

(15) $$Rec=\frac{TP}{TP+FN}\times 100\%$$

(16) $$F1=\frac{2\times Pre\times Rec}{Pre+Rec}\times 100\%$$

where TP is true positive, TN is true negative, FP is false positive, and FN is false negative.

### Experimental Results and Analyses of Improved Models

**Figure 9:**
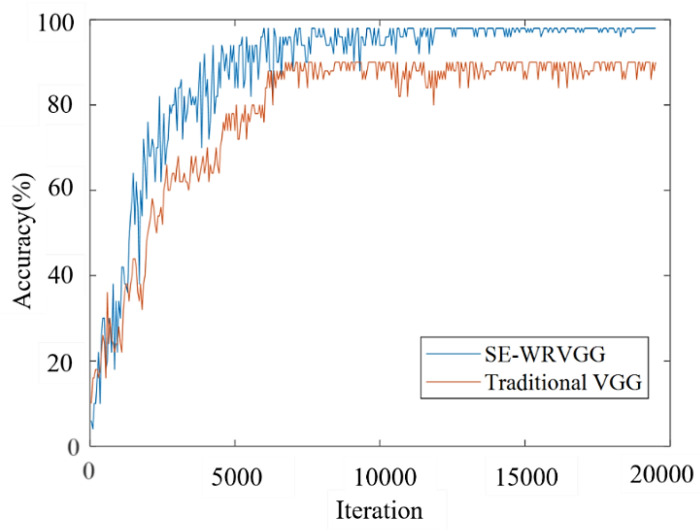
Comparison of identification results.

(1) Comparison of the abilities of the improved model and the original model. In order to verify that the SE-WRVGG model is superior to the original model, we compare and analyze the accuracy of the two training processes, and the results are shown in Figure 9.

As can be seen from Figure 9, the SE-WRVGG model starts to converge when iterated to 9000 times, and the SE-WRVGG model tends to be stable when iterated to 15,000 times, at which time the recognition effect is the best, with a recognition rate of 98.65%. In contrast, the traditional VGG model converges when iterated 15,000 times, with a recognition rate of 89.63%, so the recognition accuracy of the SE-WRVGG model is better than the traditional VGG model in terms of recognition accuracy.

**Figure 10:**
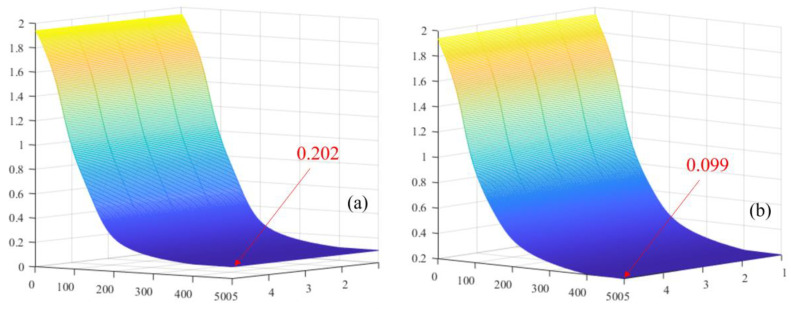
Comparison of loss value results. (**a**) SE-WRVGG model loss value, (**b**) traditional VGG model loss value.

Not only that, but we also extracted the loss values of the SE-WRVGG model and the traditional VGG model for comparison, and the comparison results are shown in Figure 10. 

From Figure 10, it can be seen that when the number of iterations is 14,900 times, the SE-WRVGG model tends to converge. When the iteration is 19,000 times, the loss value of the SE-WRVGG model does not decrease, and the accuracy tends to flatten out. At this time, the model is the most effective, with the loss value of 0.099, while the loss value of the traditional VGG model iterates to the end of the loss value of 0.202, so it can be seen that this paper’s SE-WRVGG model has better convergence than the traditional VGG model.

(2) The effect of the attention mechanism on accuracy. To verify the effect of the attention mechanism on the performance of the WRVGG model, the wire rope damage dataset was input into the SE-WRVGG model and the WRVGG model for comparative analysis, and the same algorithm was used to conduct five experiments randomly, and the average value was taken as the result. Table 1 shows the recognition results of different models.

As can be seen from Table 1, the average recognition rate of the WRVGG model is 89.49%, while the average recognition rate of the WRVGG model algorithm based on the attention mechanism is 98.84%, which is significantly higher than that of the WRVGG model, which fully proves that the introduction of the attention mechanism can effectively improve the recognition performance of the VGG model.

(3) The effect of improved network structure on training time and recognition results. In this paper, the pooling layer and the fully connected layer of the VGG model are improved, and at the same time, the BN normalization layer is added for the normalization unification process. In order to verify the superiority of the improved network structure, the training time results and recognition results of the improved VGG model and the traditional VGG model as a whole are compared, and the steel wire rope damage dataset is inputted into the improved VGG model and the traditional VGG model to be compared, and randomly conducted five experiments and took the average value as the final result. The training time results are shown in Table 2.

From Table 2, it can be seen that the average training time of the traditional VGG algorithm is 8328.59 s, while the average training time of the SE-WRVGG algorithm is 6089.38 s, which is 26.89% less than the training time of the VGG model. Thus, the model in this paper has a lower training time cost, which further proves the superiority of the SE-WRVGG algorithm.

(4) The effect of the cross-entropy loss function with joint uniform distribution on the recognition effect. In order to compare the effectiveness of the optimized function, this paper carried out a comparison experiment between the traditional loss function and the optimized loss function. The wire rope damage dataset is input into the SE-WRVGG model, and SE-VGG model for comparative analysis, and the same algorithm is randomly conducted for five experiments, and the average value is taken as the final result. Table 3 shows the identification results of different models.

As can be seen from Table 3, the average recognition rate of the SE-VGG network is 92.7%, while the average recognition rate of the SE-WRVGG algorithm, which introduces the joint uniformly distributed cross-entropy, is 98.84%, which is significantly higher than that of the SE-VGG network model, which proves that the joint uniformly distributed cross-entropy loss function proposed in this paper can alleviate the overfitting problem caused by the traditional adoption of the cross entropy loss function, performs well, and can greatly improve the recognition rate.

(5) Comparison of the improved model with other models. In order to further verify the recognition performance of the SE-WRVGG network model, the ALEXNET network model, the GOOGLENET network model, the RESNET50 model, and the CNN-LSTM network model are introduced for comparison, and the wire rope damage dataset is inputted into the five methods, and at the end of the iteration, the recognition results are obtained as shown in Table 4.

As shown in Table 4, the recognition rate of the SE-WRVGG model is 98.84%, the ALEXNET algorithm is 91.5%, the GOOGLENET algorithm is 90.27%, the RESNET50 model is 88.79%, and the CNN-LSTM algorithm is 91.73%. It can be seen from the comparison that the recognition rate of the SE-WRVGG model’s recognition rate is 7.34%, 8.57%, 10.05%, and 7.11% higher than the other three models, respectively. Not only that, the SE-WRVGG network model not only has a great improvement in accuracy but also has a more obvious improvement effect in precision rate, recall rate, and F1 value, which fully shows the superiority of the model in this study, and has more advantages in the recognition of internal damage of steel wire ropes compared with other models.

In summary, the SE-WRVGG model is superior for the identification of internal damage identification to wire ropes.

## 5. Conclusions

In this study, an improved VGG model based on the attention mechanism is proposed as a method for internal damage recognition of steel wire rope, which obtains the wire rope damage image dataset by converting the damage signal into time-frequency spectrograms and, at the same time, improves the traditional VGG model, which greatly improves the recognition rate. The specific innovation is to introduce the attention mechanism to improve the VGG network model, assign weights to the important features, and increase the weight of effective features in the VGG network. According to the experimental results, the VGG model based on the attention mechanism improves the recognition rate by 9.35% compared with the traditional VGG model, which effectively improves the accuracy of recognition. The BN batch normalization layer is added, the structure can process the data in a unified way, which is convenient for the model operation and convergence, at the same time removing the first fully connected layer and the second fully connected layer, and the maximum pooling layer is replaced with global hybrid pooling, which solves the problem of many parameters of the original network to a certain extent and reduces the training time of the model, and the training time is reduced by 26.89%. The joint sample uniformly distributed cross-entropy is used as the loss function to solve the overfitting problem and further improve the recognition rate. According to the experimental results, the recognition rate of the SE-WRVGG algorithm with the introduction of the joint uniformly distributed cross-entropy increased by 6.14% compared with the SE-VGG algorithm, which further improved the recognition effect. By comparing with other network models, the present model has a high recognition rate, which is higher than that of the ALEXNET network model, the GOOGLENET network model, the RESNET50 model, and the CNN-LSTM network model, respectively, 7.34%, 8.57%, 10.05%, and 7.11%, proving that the model in this paper has some superiority.

## Figures and Tables

**Table 1:** Results of the identification of models by the attention mechanism.

Identification Methods	Identification Accuracy (%)					Average Identification Accuracy (%)
	1	2	3	4	5	
WRVGG	89.63	89.63	88.91	89.63	89.63	89.49
SE-WRVGG	98.65	99.12	98.65	98.65	99.12	98.84

**Table 2:** Effect of improved network structure on training time.

Identification Methods	Training Time (s)					Average Training Time (s)
	1	2	3	4	5	
VGG	8258.45	8467.31	8321.73	8351.62	8243.86	8328.59
SE-WRVGG	6021.54	6186.11	6092.36	6113.27	6033.64	6089.38

**Table 3:** Effect of improved network structure on recognition results.

Identification Methods	Identification Accuracy (%)					Average Identification Accuracy (%)
	1	2	3	4	5	
SE-VGG	92.72	92.69	92.69	92.69	92.72	92.7
SE-WRVGG	98.65	99.12	98.65	98.65	99.12	98.84

**Table 4:** Comparison of recognition effects of different models.

Serial Number	Identification Methods	Acc (%)	Pre (%)	Rec (%)	F1 (%)
1	SE-WRVGG	98.84	98.25	97.91	98.08
2	ALEXNET	91.5	91.17	91.23	91.19
3	GOOGLENET	90.27	90.16	91.15	90.65
4	RESNET50	88.79	87.64	88.05	87.84
5	CNN-LSTM	91.73	91.35	91.82	91.58

## Data Availability

The data presented in this study are available on request from the corresponding author.

## Abbreviations

VGG model	a very deep convolutional network for large model recognition.
STFT method	short-time Fourier transform method.
SE Attention Mechanism Module	Channel Attention Module.
BN layer	batch normalization layer.
GWO-SVM algorithm	grey wolf optimization algorithm optimized support vector machine algorithm.
HOG algorithm	an algorithm for extracting histogram features of directional gradients.
DCNN networks	dynamic convolutional neural networks.
AUTO-CNN network	autonomous convolutional neural network.
ALEXNET network model	deep convolutional neural network.
GOOGLENET network model	a deep network structure developed by Google.
RESNET50 model	deep residual network.
CNN-LSTM network model	convolutional neural network combined with long-short-term memory network.
